# Revealing a cancer-associated fibroblast-based risk signature for pancreatic adenocarcinoma through single-cell and bulk RNA-seq analysis

**DOI:** 10.18632/aging.206043

**Published:** 2024-09-26

**Authors:** Jing Ma, Zhinan Chen, Limin Hou

**Affiliations:** 1 Department of Emergency Surgery, The First Affiliated Hospital of Harbin Medical University, Harbin, China

**Keywords:** cancer-associated fibroblasts, pancreatic adenocarcinoma, risk signature, immunotherapy, nomogram

## Abstract

Purpose: Proliferation of stromal connective tissue is a hallmark of pancreatic adenocarcinoma (PAAD). The engagement of activated cancer-associated fibroblasts (CAFs) contributes to the progression of PAAD through their involvement in tumor fibrogenesis. However, the prognostic significance of CAF-based risk signature in PAAD has not been explored.

Methods: The single-cell RNA sequencing (scRNA-seq) data sourced from GSE155698 within the Gene Expression Omnibus (GEO) database was supplemented by bulk RNA sequencing data from The Cancer Genome Atlas (TCGA) and microarray data retrieved from the GEO database. The scRNA-seq data underwent processing via the Seurat package to identify distinct CAF clusters utilizing specific CAF markers. Differential gene expression analysis between normal and tumor samples was conducted within the TCGA-PAAD cohort. Univariate Cox regression analysis pinpointed genes associated with CAF clusters, identifying prognostic CAF-related genes. These genes were utilized in LASSO regression to craft a predictive risk signature. Subsequently, integrating clinicopathological traits and the risk signature, a nomogram model was constructed.

Results: Our scRNA-seq analysis unveiled four distinct CAF clusters in PAAD, with two linked to PAAD prognosis. Among 207 identified DEGs, 148 exhibited significant correlation with these CAF clusters, forming the basis of a seven-gene risk signature. This signature emerged as an independent predictor in multivariate analysis for PAAD and demonstrated predictive efficacy in immunotherapeutic outcomes. Additionally, a novel nomogram, integrating age and the CAF-based risk signature, exhibited robust predictability and reliability in prognosticating PAAD. Moreover, the risk signature displayed substantial correlations with stromal and immune scores, as well as specific immune cell types.

Conclusions: The prognosis of PAAD can be accurately predicted using the CAF-based risk signature, and a thorough analysis of the PAAD CAF signature may aid in deciphering the patient’s immunotherapy response and presenting fresh cancer treatment options.

## INTRODUCTION

Given its elevated aggressiveness and unfavorable prognosis, pancreatic adenocarcinoma (PAAD) represents a substantial threat to human life and well-being. Epidemiological studies show that pancreatic cancer is the fourth most lethal form of cancer, while being the 12^th^ most frequent tumor worldwide [1, 2]. Pancreatic ductal adenocarcinoma (PDAC) comprises the majority of PAAD cases, displaying low responsiveness to radiotherapy, a challenging resection rate, and a notably poor prognosis, with a 5-year survival rate of less than 7% [1, 3]. The progression of PAAD from genetic mutation to abnormal cell proliferation and precancerous lesions, culminating in early carcinoma, spans an approximate duration of 5-20 years. However, a PAAD tumor can rapidly progress from a tiny lump to an advanced stage in as little as 6 to 20 months. Moreover, the asymptomatic progression of pancreatic tumors often leads to the diagnosis of pancreatic cancer at advanced stages in most individuals. Therefore, early identification and prediction of disease progression in PAAD are of significant clinical importance.

Pancreatic cancer therapy and patient outcomes can be greatly improved by a deeper understanding of the pathogenesis and development of PAAD, as well as the identification of novel molecular targets for the disease. Rapid advances in omics technology over the past few decades have greatly aided our understanding of the molecular pathogenesis of PAAD [4, 5]. Akin to numerous malignancies, PAAD intricately engages multifaceted molecular cascades. Presently, researchers have adeptly fashioned a variety of intricate polygenic prognostic risk models leveraging advanced bioinformatics methodologies. Gene signatures obtained from omic data have been developed to forecast PAAD clinical outcomes [6, 7]. A multigene prognostic model serves as a valuable tool for assessing patients’ overall survival duration and risk of recurrence. It enables the identification of high-risk individuals with a poor prognosis, facilitating timely and systematic intervention. Conversely, it allows for the appropriate avoidance of unnecessary treatment burden among low-risk patients. In light of this, unique multigene signatures are required for predicting PAAD outcomes and recurrence.

Throughout the progression of cancer, the microenvironment of PAAD cells is conducive to their survival, proliferation, and distant metastasis [8]. Research has underscored the fundamental importance of the ongoing interaction between cancer cells and stromal cells in the process of cancer development and progression [9]. Cancer-associated fibroblasts (CAFs) are comparatively prevalent stromal cell components that have been observed in a variety of malignancies, including breast cancer, prostate cancer, and hepatocellular carcinoma, and their communications with cancer cells have been shown to be essential for cancer development [10–12]. Noteworthy is the possibility that certain biological traits, including pronounced interstitial fibrosis, are linked to the poor prognosis of pancreatic cancer [13]. Tumor cell proliferation, metastasis, and chemotherapy resistance may all be influenced by CAFs due to the secretion of various growth factors, chemokines, and cytokines, as well as the degradation of extracellular matrix proteins [14, 15]. The tumor-promoting properties of CAFs might be maintained even in the absence of cancer cells [16]. Subsequently, blocking CAF-derived effects or inhibiting CAF-secreted components that promote tumor formation and progression has emerged as a viable method for PAAD treatments.

Previous study has identified five new CAFs subclusters based on the marker genes with distinct signaling patterns and immune status in PAAD using single-cell sequence transcriptomic data [17]. However, there is still a lack of knowledge on how PAAD prognosis and immunotherapy response are connected to the features of CAF on a systemic level. Transcriptomic and single-cell RNA-sequencing (scRNA-seq) data pertaining to PAAD were obtained from the GEO and TCGA databases. We classified CAFs into distinct subclusters and established a CAF-based risk signature for PAAD. To enhance the clinical applicability of CAF characteristics in predicting PAAD, we devised a distinctive nomogram that integrates the CAF-based risk signature alongside clinicopathological variables. Finally, the immunological landscape and response to immunotherapy that underlie the CAF-based signature were further explored, and the clinical significance was established. Our findings could offer fresh perspectives on the pathogenesis of PAAD, paving the way for more individualized therapies and better results for PAAD patients.

## MATERIALS AND METHODS

### Data collection and processing

We obtained scRNA-seq data (GSE155698) from the Gene Expression Omnibus (GEO) database, encompassing pancreatic cancer tumor samples from 16 patients alongside 3 adjacent normal pancreas samples. Scanning the scRNA-seq data involved an initial screening of single cells, ensuring each gene was expressed in at least three cells, while maintaining a minimum threshold of 250 expressed genes per cell. To determine the relative abundance of mitochondria and rRNA, the Seurat R package was employed [18]. Each single cell was then configured to express at least 6000 genes and have a UMI greater than 100 for further screening. After everything was filtered out, 50,527 cells were left. Utilizing The Cancer Genome Atlas (TCGA) database, we accessed transcriptome data, single-nucleotide variant (SNV) data, copy number variant (CNV) data, and associated clinical information for PAAD. Additionally, expression matrices from GSE78229 and GSE85916 were acquired from the GEO database for validation purposes.

### Clustering of CAFs

In an effort to better describe the CAF signature, we redeployed the scRNA-seq data of PAAD. After log normalization, cells that had either over 6000 or under 250 genes being actively expressed were filtered out. We addressed batch effects and conducted non-linear dimensional reduction. Using the FindNeighbors and FindClusters functions, individual cells were partitioned into separate subgroups. Subsequently, this subdivision was visualized via t-distributed stochastic neighbor embedding (t-SNE) dimensional reduction. Six genes were used to annotate fibroblasts, including ACTA2, FAP, PDGFRB, NOTCH3, DCN, and COL1A2. Using the same approach, FindNeighbors and FindClusters were used to re-cluster the fibroblasts, which was visualized with the TSNE dimensionality reduction. We utilized the FindAllMarkers function to identify specific marker genes within each CAF cluster. Subsequently, functional enrichments were conducted on these CAF marker genes using the Kyoto Encyclopedia of Genes and Genomes (KEGG) analysis via the clusterProfiler package [19]. The CopyKAT R package was used to distinguish tumor cells from normal cells by analyzing CNV features among CAFs clusters [20].

### Identification of prognostic genes

Differentially expressed genes (DEGs) between 177 pancreatic tumor samples and 4 normal tissue samples were first identified in the TCGA-PAAD cohort. We then analyzed the DEGs for CAF clusters to determine which genes are most strongly correlated with CAFs (*P* < 0.001, cor > 0.4). Further prognosis-associated genes were identified through univariate Cox regression analysis, considering a significance threshold of P < 0.05. To reduce the pool of prognostic genes, initial analysis involved least absolute shrinkage and selection operator (LASSO) Cox regression, yielding seven genes. Subsequently, a multivariate Cox regression employing stepwise regression constructed the risk signature, calculating coefficients via the formula: risk score = Σβi*Expi. Patients were stratified into high- or low-risk groups based on their risk scores. An evaluation of the risk signature’s predictive performance was conducted using receiver operating characteristic (ROC) analysis. Analyses in the validation cohorts were carried out in a similar manner.

### Nomogram construction

To create a clinically applicable nomogram model, we initiated univariate and multivariate Cox regression analyses incorporating the risk signature and clinicopathological factors such as age, gender, stage, and race. The resulting multivariate Cox model, including variables with *P* < 0.05, was utilized to devise a prognostic nomogram for PAAD using the rms package [21]. The prediction efficacy of the model was assessed by the calibration curve and the reliability was assessed using decision curve analysis (DCA).

### Immune infiltration analysis

The immune and stromal scores of the tumor microenvironment (TME) were calculated using the ESTIMATE algorithm and the relative abundance of infiltrating immune cells in the TME was evaluated by the CIBERSORT and MCPcounter algorithms [22].

### Evaluation of responses to immunotherapy

We collected clinical and transcriptomic data of PAAD patients treated with anti-PD-L1 therapies from the IMvigor210 cohort available at http://research-pub.gene.com/IMvigor210CoreBiologies. Furthermore, GSE78220 comprises pre-treatment melanoma samples that underwent anti-PD-1 checkpoint blockade (ICB) immunotherapy. This transcriptomic dataset can be accessed to assess the potential utility of the risk signature in predicting response to ICB therapy [23].

### Statistical analysis

R software (version 3.6.3) was used for all statistical testing. The Pearson or Spearman correlation method was used for correlation analysis. The Wilcoxon test was used to examine the differences between the two groups. K-M curves with Log-rank testing were used to compare survival times across groups. Statistical significance was assumed at a *P*-value of less than 0.05.

### Data availability statement

Publicly available datasets were analyzed in this study. The data can be found in GEO and TCGA databases.

## RESULTS

### Identification of four CAF-related clusters for PAAD

The single-cell RNA-seq dataset GSE155698 was employed to identify the CAF subpopulations in PAAD, which included 17 PAAD tumor samples from 16 patients (Patient 11 had samples A and B) and 3 adjacent normal pancreas samples. Following preliminary screening, a total of 50,527 cells were collected (Supplementary Figure 1A, 1B). Supplementary Figure 1C displayed the comprehensive outcomes of the data preprocessing, which demonstrated a high quality control for the subsequent analysis. The UMAP plot displayed the distributions of the 20 samples after removal of the batch effect (Supplementary Figure 1D). After log-normalization and reducing dimensions, a total of 31 distinct subpopulations were identifiable (Supplementary Figure 1E). Of note, six CAF marker genes, including ACTA2, FAP, PDGFRB, NOTCH3, DCN, and COL1A2, were observed to be highly expressed in the subpopulation 10 and 17, which were therefore considered as CAF populations (Supplementary Figure 1F).

**Figure 1:**
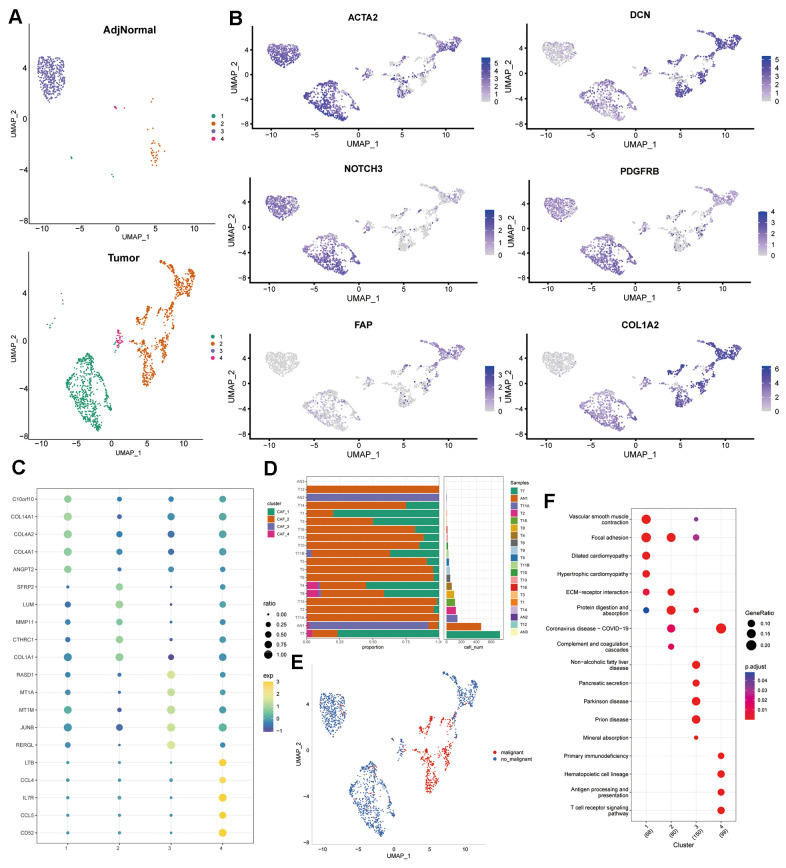
**Profiling of CAF subpopulations.** (**A**) UMAP plot displaying the distribution of four distinct CAF subpopulations post-clustering. (**B**) UMAP plot illustrating the expression of CAF marker genes (ACTA2, FAP, PDGFRB, NOTCH3, DCN, and COL1A2). (**C**) Dot plot showcasing the top 5 marker gene expressions across the four CAF clusters. (**D**) Relative proportions and cell numbers within each sample for the four CAF clusters. (**E**) UMAP plot delineating the distribution between malignant and non-malignant cells. (**F**) KEGG enrichment analysis of DEGs observed across the four CAF clusters.

Four CAF clusters were then identified after extracting cells from the two CAF populations (subpopulation 10 and 17) using the same clustering approach for dimensionality reduction (Figure 1A). The varying expression of the six CAF marker genes within the four CAF clusters suggests distinct marker gene expressions among different subpopulations (Figure 1B). A total of 860 DEGs were recognized within the four CAF clusters, and Figure 1C illustrates the expression patterns of the top 5 DEGs within these clusters, serving as the designated marker genes. The proportions of the four clusters in each sample were shown in Figure 1D. As determined by CNV characteristics, the four CAF clusters contained 1415 normal cells and 537 tumor cells (Figure 1E). Furthermore, KEGG enrichment analysis showed that DEGs were enriched in multiple pathways across the four clusters, including vascular smooth muscle contraction, focal adhesion, dilated cardiomyopathy, hypertrophic cardiomyopathy, ECM-receptor interaction, and Protein digestion and absorption (Figure 1F).

### Expression of distinct signaling patterns in CAF clusters

**Figure 2:**
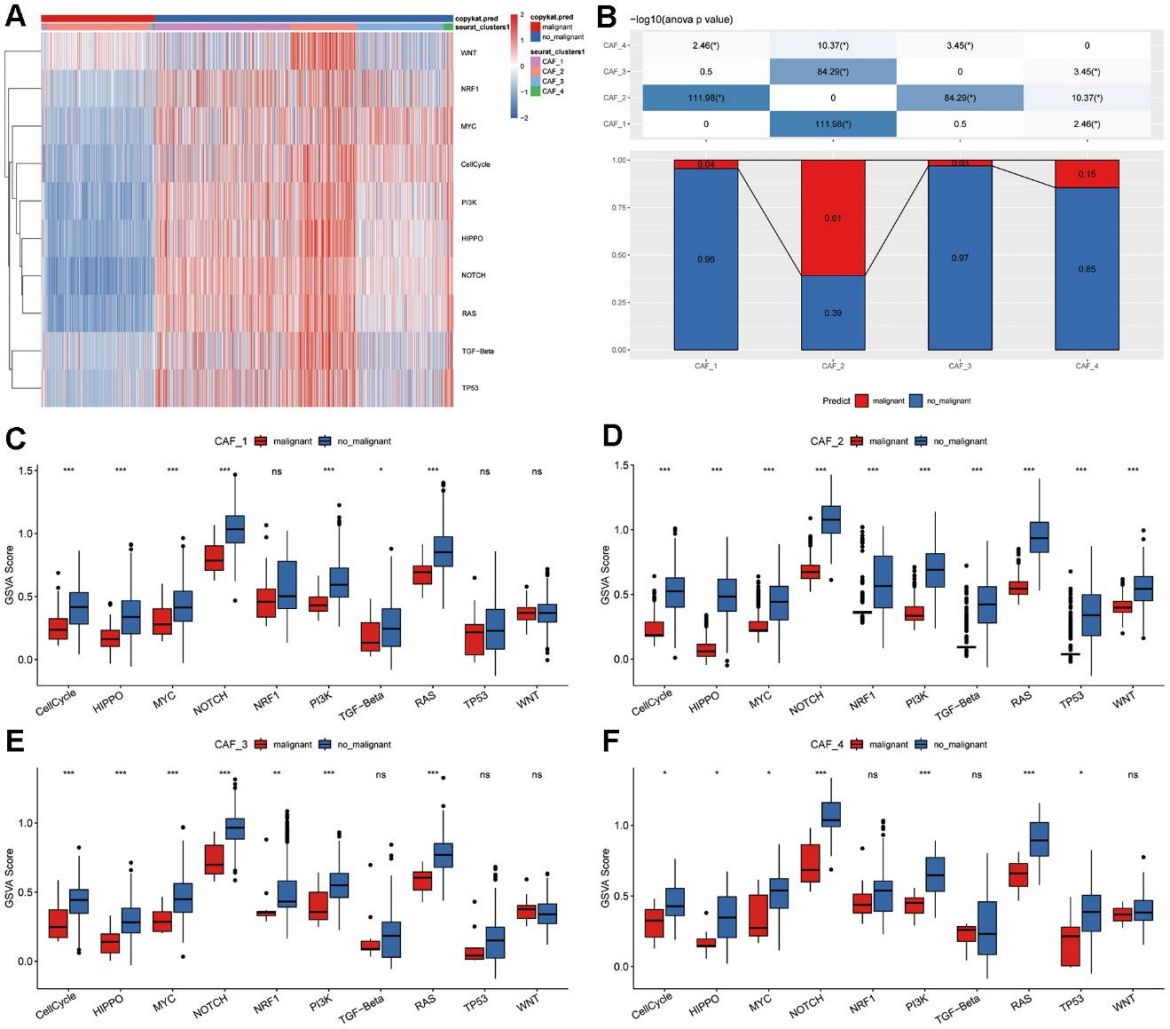
**Tumor-related pathway characteristics in CAF clusters.** (**A**) Heatmap illustrating GSVA scores for ten tumor-related pathways enriched in both malignant and non-malignant CAF cells. (**B**) Comparison of malignant and non-malignant cell proportions across different CAF clusters. Comparative analysis of GSVA scores for ten tumor-related pathways between malignant and non-malignant cells within (**C**) CAF_1, (**D**) CAF_2, (**E**) CAF_3, and (**F**) CAF_4 clusters. *P < 0.05; **P < 0.01; ***P < 0.001; ns, not statistically significant.

To investigate the potential role of CAF clusters in tumorigenesis, the expression of ten tumor-related pathways were evaluated in the four CAF clusters, including WNT, NRF1, MYC, CellCycle, PI3K, HIPPO, NOTCH, RAS, TGF-Beta, and TP53 signaling pathways. The GSVA scores of these pathways across the four CAF clusters were presented in the heatmap (Figure 2A). CAF_2 cluster exhibited notably higher proportions of malignant cells compared to the other three clusters (Figure 2B). Moreover, the CAF_4 cluster displayed a higher proportion of malignant cells compared to both CAF_1 and CAF_3 clusters. Furthermore, we conducted a comparison of GSVA scores for ten tumor-related pathways between malignant and non-malignant cells within each CAF cluster (Figure 2C–2F). Non-malignant cells had considerably higher GSVA scores for most signaling pathways in all CAF clusters, while minor variations among groups were observed.

### Associations between CAF clusters and clinical characteristics of PAAD patients

To investigate potential associations between CAF clusters and clinical characteristics of PAAD patients, the ssGSEA scores for specific marker genes—C10orf10, COL14A1, COL4A2, COL4A1, and ANGPT2 for CAF_1; SFRP2, LUM, MMP11, CTHRC1, and COL1A1 for CAF_2; RASD1, MT1A, MT1M, JUNB, and RERGL for CAF_3; and LTB, CCL4, IL7R, CCL5, and CD52 for CAF_4 (as identified in Figure 1C)—were calculated within each CAF cluster based on the TCGA cohort. Supplementary Figure 2A demonstrated that the CAF_4 cluster displayed notably lower scores in tumor samples in comparison to normal samples, with no statistically significant differences observed in the remaining CAF clusters. Following this, to assess the prognostic significance, the PAAD samples within the TCGA cohort were stratified based on high and low CAF scores. There was no correlation between CAF_1 and CAF_3 clusters and PAAD prognosis, while the CAF_2 and CAF_4 clusters fared better for low-CAF score samples than high-CAF score samples. (Supplementary Figure 2B). Next, we set out to establish the clinical correlations of CAF clusters. When PAAD patients were segmented into two categories based on gender (female vs. male), the disparity of CAF_1 score, but not other CAF clusters, was observed (Supplementary Figure 2C–2F). However, no clinical associations between CAF clusters and age were discovered. Furthermore, CAF_3 and CAF_4 scores were shown to be considerably higher in white persons compared to Asian people, whereas no associations were found between CAF scores and pathological stages.

### Construction of CAF-related risk signature

**Figure 3:**
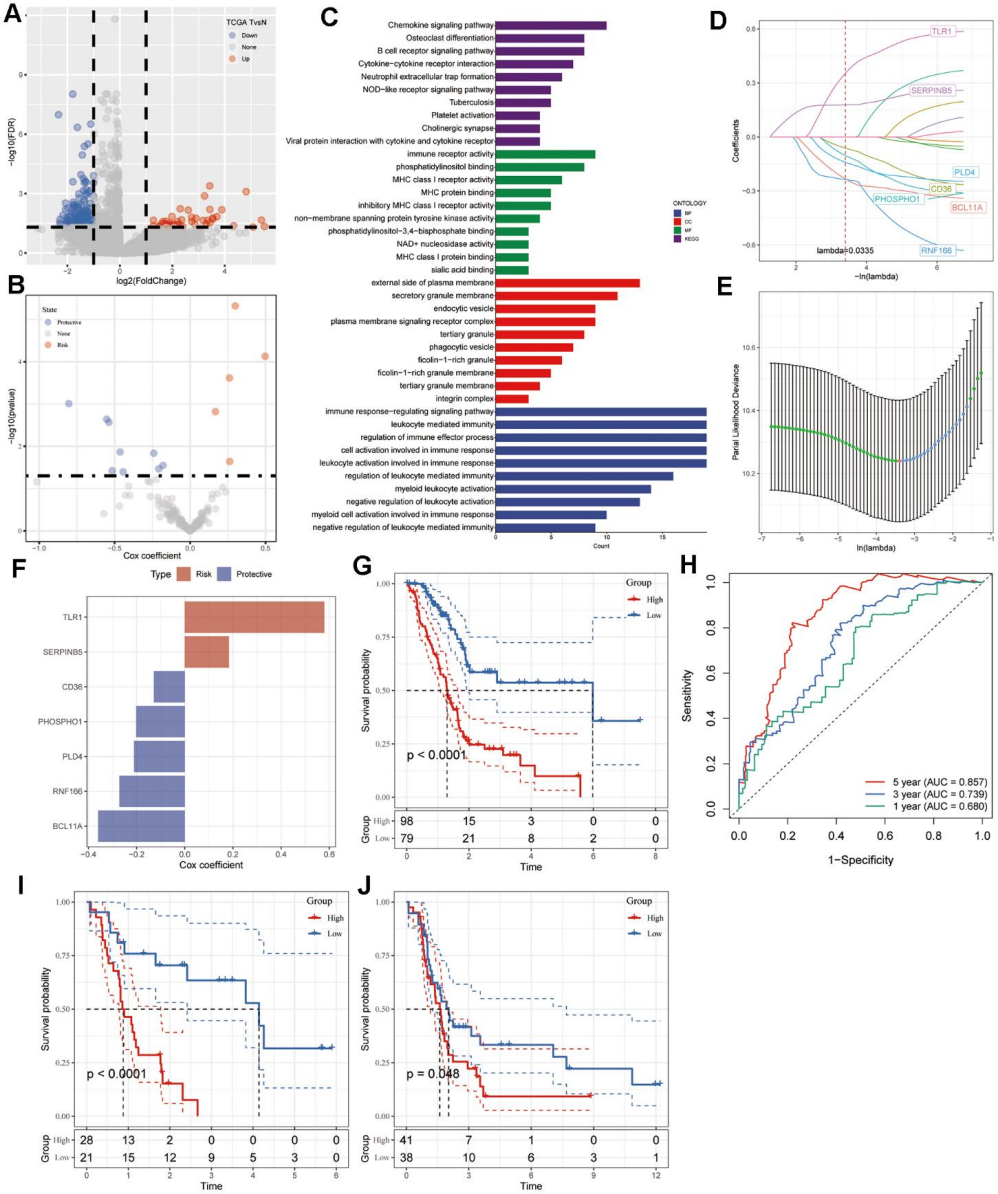
**Identification of CAF-associated hub genes with prognostic significance.** (**A**) Volcano plot illustrating DEGs between tumor and normal tissues in the TCGA-PAAD cohort. (**B**) Volcano plot showcasing prognosis-related genes identified through univariate Cox regression analysis. (**C**) Functional enrichment analyses encompassing GO (BP, CC, and MF) and KEGG analyses of CAF-related DEGs. (**D**) Trajectory plot depicting each independent variable with lambda in the LASSO model for PAAD. (**E**) LASSO coefficient profiles highlighting the seven genes in PAAD. The plot shows coefficient profiles against the log (lambda) sequence. (**F**) Multivariate Cox coefficients for each gene in the risk signature. (**G**) Kaplan-Meier curves illustrating the risk model constructed using the seven genes in the TCGA-PAAD cohort. (**H**) ROC curves displaying the risk model constructed with the seven genes in the TCGA-PAAD cohort. Kaplan-Meier curves of the risk model constructed with the seven genes in the validation datasets (**I**) GSE78229 and (**J**) GSE85916.

We initiated the identification of DEGs by comparing 177 tumor samples with 4 normal samples in the TCGA-PAAD cohort to establish a risk signature. As depicted in Figure 3A, a total of 207 DEGs were discerned, comprising 47 up-regulated and 160 down-regulated genes. Among these, 148 genes displayed significant associations with CAF clusters. Subsequently, univariate Cox regression analysis was conducted on these 148 genes to assess their prognostic significance. Out of these, 14 genes exhibited prognostic values; 5 genes were identified as risk-associated, while 9 genes were deemed protective (Figure 3B). Functional annotations for the 148 genes were presented in Figure 3C. The significantly enriched biological process terms were immune response-regulating signaling pathway, leukocyte mediated immunity, regulation of immune effector process, cell activation involved in immune response, and leukocyte activation involved in immune response. In the cellular component part, genes were particularly enriched in external side of plasma membrane, secretory granule membrane, endocytic vesicle, plasma membrane signaling receptor complex, and tertiary granule. Meanwhile, immune receptor activity, phosphatidylinositol binding, MHC class I receptor activity, MHC protein binding, and inhibitory MHC class I receptor activity were mainly enriched in the molecular function group. In addition, integrated DEGs were mainly involved in Chemokine signaling pathway, Osteoclast differentiation, B cell receptor signaling pathway, Cytokine-cytokine receptor interaction, and Neutrophil extracellular trap formation in KEGG pathway analysis. Subsequently, we utilized LASSO regression analysis to further screen 14 prognostic genes and finally obtained seven genes as lambda = 0.0335 in the model, including Toll-Like Receptor 1 (TLR1), Serpin Family B Member 5 (SERPINB5), Phospholipase D Family Member 4 (PLD4), CD36 Molecule (CD36), Phosphocholine Phosphatase 1 (PHOSPHO1), BCL11 Transcription Factor A (BCL11A), Ring Finger Protein 166 (RNF166) (Figure 3D, 3E). We utilized multivariate Cox regression analysis employing a stepwise regression method to establish the risk signature. The resulting final seven-gene signature formula is represented as follows: RiskScore = -0.129 * CD36 - 0.202 * PHOSPHO1 - 0.211 * PLD4 + 0.58 * TLR1 + 0.183 * SERPINB5 - 0.36 * BCL11A - 0.272 * RNF166 (Figure 3F). Following z-mean normalization, the risk score was computed for each sample and categorized into high- and low-risk groups. Kaplan-Meier survival analysis conducted on the TCGA cohort revealed notably poorer survival outcomes among individuals in the high-risk category compared to those in the low-risk group (Figure 3G). In the TCGA cohort, the model displayed varying 1- to 5-year AUC values ranging from 0.680 to 0.857 (Figure 3H). Figure 3I, 3J displayed the survival curves for validation datasets GSE78229 and GSE85916. The findings indicate notably poorer prognosis among patients in the high-risk group.

### Development of nomogram based on CAF-related gene signature in PAAD patients

**Figure 4:**
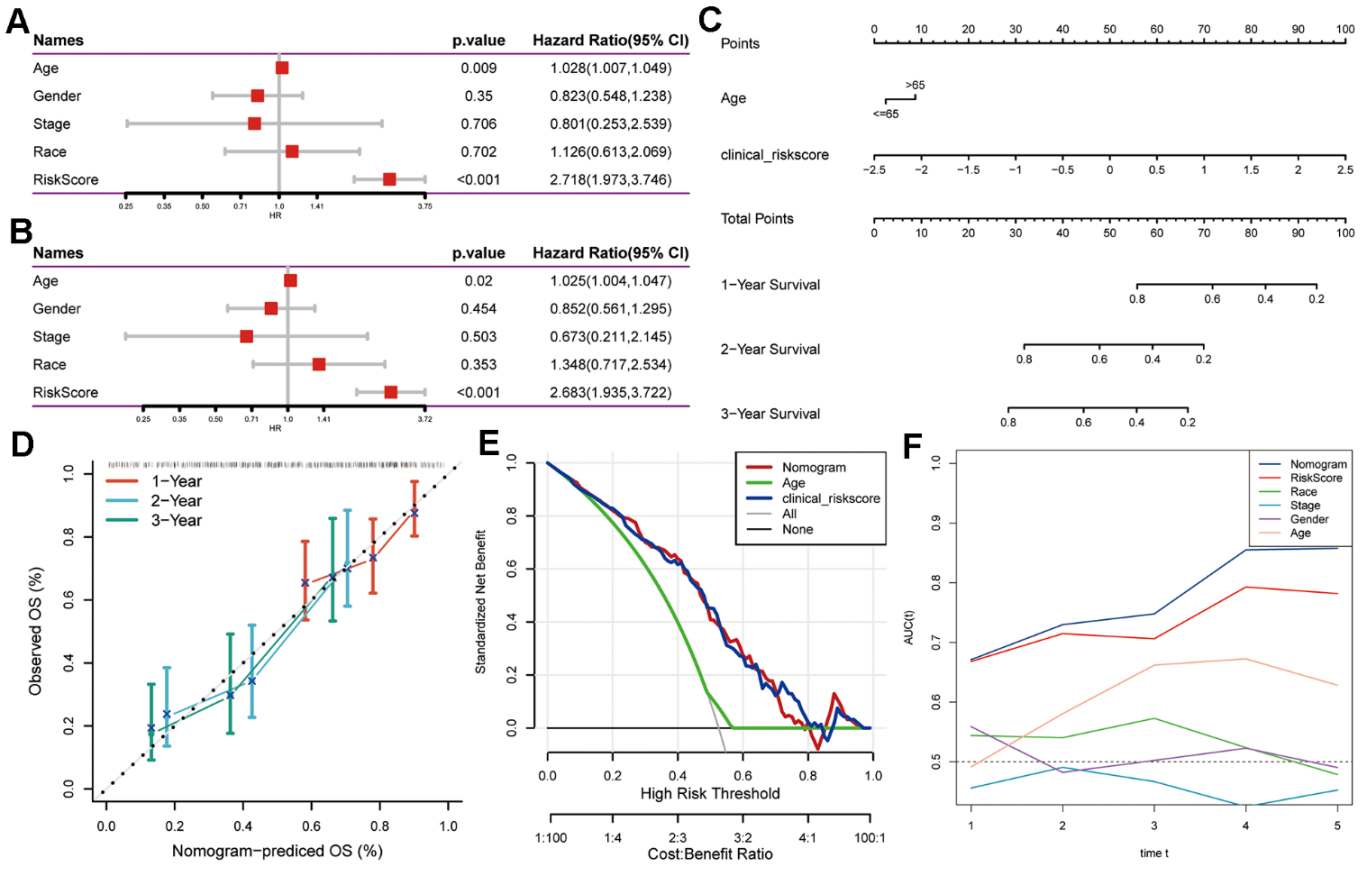
**Creation of a nomogram using CAF-related gene signature for PAAD prognostication.** (**A**) Univariate and (**B**) multivariate Cox regression analyses involving risk score and clinicopathological characteristics. (**C**) Development of a nomogram model amalgamating age and risk score. (**D**) Calibration plots showcasing the prediction accuracy for 1-, 2-, and 3-year survival probabilities. (**E**) Decision curve analysis illustrating the nomogram’s utility. (**F**) Time-ROC curve analysis comparing the predictive performance of the nomogram against other factors.

Utilizing both univariate and multivariate Cox regression analyses, we amalgamated clinicopathological characteristics to enhance the predictive capability of the CAF-related gene signature. In the initial phase of our investigation, a univariate analysis highlighted age [hazard ratio (HR) = 1.028, 95% confidence interval (CI): 1.007 – 1.049, *P* = 0.009] and risk score (HR = 2.718, 95% CI: 1.973 – 3.746, *P* < 0.001) as notably linked to PAAD patient survival (Figure 4A). Subsequent multivariate Cox regression analysis confirmed the risk score (HR = 2.683, 95% CI: 1.935 – 3.722, P < 0.001) as an independent prognostic indicator for PAAD, considering other influencing factors (Figure 4B). Consequently, we developed a visual nomogram incorporating age and risk score to predict individual survival at 1, 2, and 3 years, depicted in Figure 4C. The calibration curve illustrated satisfactory alignment between actual observations and predictions across 1-, 2-, and 3-year intervals (Figure 4D). Demonstrating superior discriminative performance for identifying high-risk patients, the DCA plot highlighted the nomogram’s superiority over age (Figure 4E). TimeROC analysis of the TCGA cohort exhibited higher AUC values for the risk score and nomogram compared to other indicators (Figure 4F).

### Mutation and pathway analyses of seven prognostic genes

**Figure 5:**
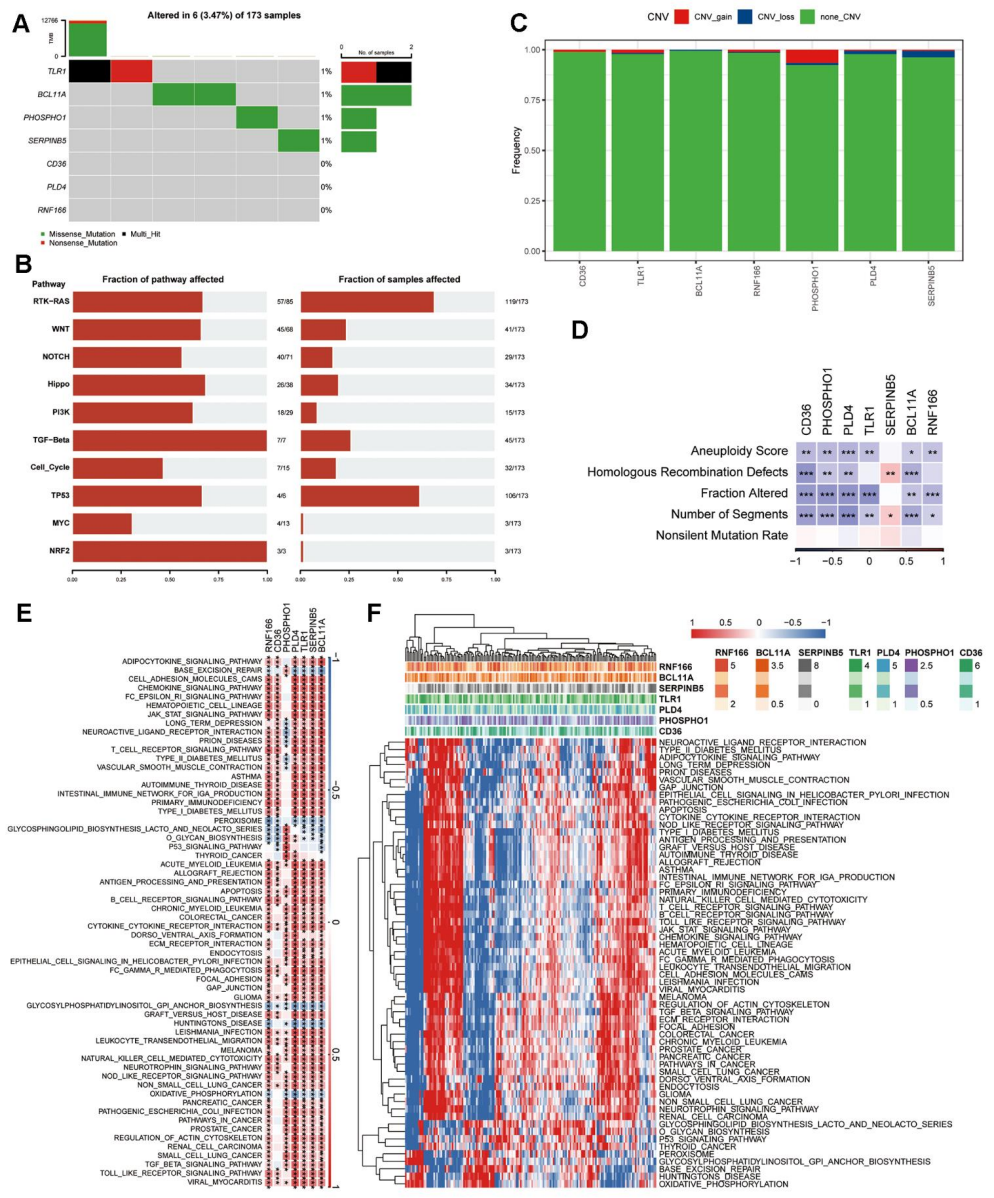
**Genetic profile of the seven genes in the risk signature.** (**A**) Waterfall diagram illustrating SNV mutations of the seven pivotal genes. (**B**) Enrichment heatmap displaying key pathways associated with SNV data in PAAD. (**C**) CNV alterations in the seven crucial genes, showcasing instances of gain, loss, and absence of alterations. (**D**) Heatmap visualizing correlations between the seven pivotal genes and Aneuploidy Score, Homologous Recombination Defects, Fraction Altered, Number of Segments, and Nonsilent Mutation Rate. (**E**) Heatmap revealing gene-pathway correlations. (**F**) Heatmap illustrating enrichment scores for pathways. *P < 0.05, **P < 0.01, ***P < 0.001.

The genetic mutations (SNV) within the seven genes forming the risk signature were subsequently examined. More samples were found to contain SNV mutations in TLR1, BCL11A, PHOSPHO1, and SERPINB5, however, no SNV mutation was found in CD36, PLD4, or RNF166 (Figure 5A). Pathway enrichment on SNV data showed that TGF-Beta and NRF2 pathways were the most affected pathways in PAAD, while more samples were involved in the RTK-RAS and TP53 pathways (Figure 5B). Within the seven genes comprising the risk signature, only a few samples displayed CNV alterations (Figure 5C). Hence, we explored the connections between these risk genes and diverse molecular indicators in PAAD, aiming to enhance our understanding of how these genes are linked to the disease. The results demonstrated that CD36, PHOSPHO1, PLD4, TLR1, BCL11A, and RNF166 were all significantly negatively correlated with Aneuploidy Score, Fraction Altered, and Number of Segments, whereas SERPINB5 exhibited notably positive correlations with Homologous Recombination Defects and Number of Segments (Figure 5D). Interestingly, the Nonsilent Mutation Rate displayed no correlation with any of these identified risk genes. Additionally, we delved into the potential pathways linked to each risk gene (Figure 5E, 5F). The heatmap revealed significant correlations of these seven genes with a total of 60 pathways, encompassing pathways like adipocytokine signaling pathway, base excision repair, and cell adhesion molecules cams.

### Relationship between immune infiltration and seven prognostic genes

We then investigated the association between the seven prognostic genes and immune infiltration. Initially, we computed the immune score, stromal score, and ESTIMATE score utilizing the ESTIMATE algorithm (Supplementary Figure 3A–3D). Our correlation analysis revealed that CD36, PHOSPHO1, PLD4, TLR1, and RNF166 exhibited positive correlations with stromal score, immune score, and ESTIMATE score. Conversely, BCL11A showed positive correlations solely with immune score and ESTIMATE score. Notably, SERPINB5 did not display significant correlations with any of these three scores. We then compared the immune score across groups that were defined by the median expression level of respective genes. For the genes CD36, PHOSPHO1, PLD4, TLR1, BCL11A, and RNF166, individuals with high expression had significantly higher immune score than those with low expression (Supplementary Figure 3E–3K). However, individuals with low SERPINB5 expression had higher immune score. We utilized the CIBERSORT algorithm to compute the abundance of infiltrating immune cells and their correlations with the seven prognostic genes. The correlation heatmap showed that CD36, PHOSPHO1, PLD4, BCL11A, and RNF166 presented a significantly positive correlation with CD8^+^ T cells, naïve and memory B cells, whereas negative correlation with CD4^+^ memory resting T cells. TLR1 was positively correlated with CD4^+^ memory activated T cells, while negatively correlated with activated NK cells and activated mast cells, similar to SERPINB5 (Supplementary Figure 3L). We further validated our results using the MCPcounter algorithm. Significantly positive correlations were observed between RNF166, TLR1, PLD4, PHOSPHO1, CD36, and infiltrating immune cells, while SERPINB5 was only positively correlated with fibroblasts, which was consistent with previous results (Supplementary Figure 3M).

### The prediction of risk signature to the efficacy of immunotherapy

**Figure 6:**
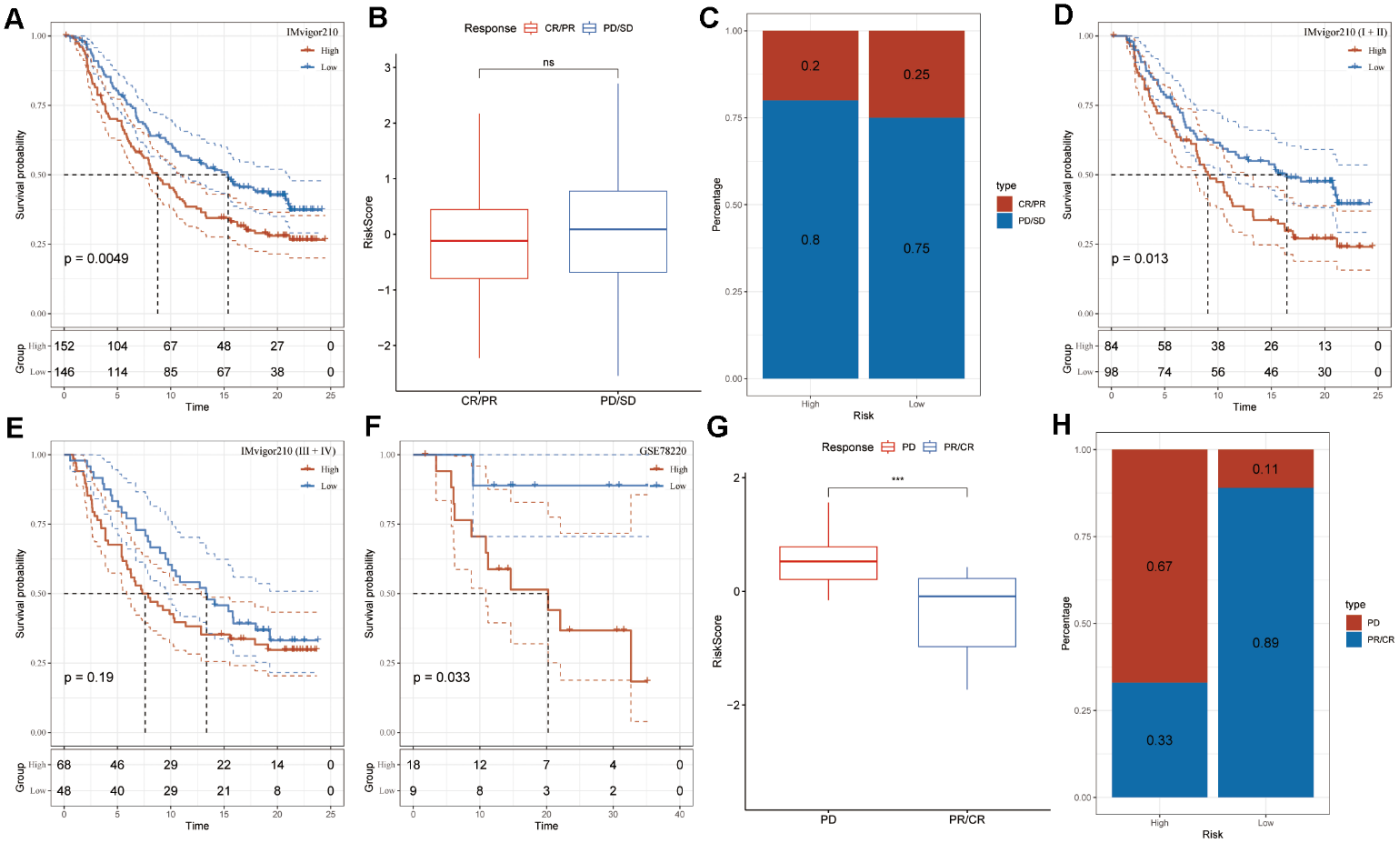
**Risk signature response to immunotherapy in IMvigor210 and GSE78220 cohorts.** (**A**) Prognostic differences among IMvigor210 cohort subgroups based on the risk score. (**B**) Variations in risk scores within IMvigor210 cohort responses to immunotherapy. (**C**) Distribution of immunotherapy responses among risk score groups in the IMvigor210 cohort. (**D**) Prognostic differences among subgroups of early-stage patients in the IMvigor210 cohort based on the risk score. (**E**) Prognostic differences among subgroups of advanced-stage patients in the IMvigor210 cohort based on the risk score. (**F**) Prognostic differences among subgroups of the GSE78220 cohort based on the risk score. (**G**) Variations in risk scores among GSE78220 cohort responses to immunotherapy. (**H**) Distribution of immunotherapy responses among risk score groups in the GSE78220 cohort.

The utilization of T-cell immunotherapy in cancer treatment has shown promise in extending patients’ survival. Consequently, we conducted an analysis on the IMvigor210 and GSE78220 cohorts to evaluate the predictive efficacy of the risk signature for immune-checkpoint therapy. Patients categorized in the IMvigor210 low-risk group exhibited better clinical outcomes and longer overall survival in comparison to those in the high-risk group (Figure 6A, P = 0.0049). However, no significant difference in risk score was observed between patients with complete/partial response (CR/PR) and those with progressive/stable disease (PD/SD), and the proportion of PD/SD patients in the high-risk group did not significantly differ from that in the low-risk group (Figure 6B, 6C). Particularly, substantial differences in survival probability were noted among various risk groups for patients diagnosed at Stages I and II (Figure 6D, P = 0.013), but not for those at Stages III and IV (Figure 6E, P = 0.19). These findings suggested higher sensitivity of the risk score for patients diagnosed at earlier stages. Similarly, patients within the low-risk group in the GSE78220 cohort displayed significantly prolonged overall survival compared to those in the high-risk group (Figure 6F, P = 0.033). Moreover, in contrast to IMvigor210, PD patients exhibited notably higher risk scores than PR/CR patients (Figure 6G), and the proportion of PD patients in the high-risk group was significantly elevated compared to the low-risk group in the GSE78220 cohort (Figure 6H). These outcomes indicate the effective predictive capacity of the risk signature in determining patient responses to immunotherapy.

## DISCUSSION

Since CAFs have been proved to be engaged in carcinogenesis by releasing numerous substances into the TME, increasing evidence has shown that this dynamic interaction between tumor cells and stroma cells leads to tumor development [9, 24]. Here, using scRNA-seq data, we systematically characterized and classified CAFs of PAAD to better understand their variety. At the conclusion of the study, we identified four distinct clusters of CAFs, each exhibiting unique characteristics potentially influencing the biological regulation of the TME. Currently, there remains a scarcity of studies focusing on the predictive relevance of CAF-secreted factors or a CAF-associated risk signature in PAAD. Zhao et al. identified five novel subcluster of CAFs based on the marker genes and both CAF-C2 and CAF-C4 subgroups had significantly negative correlation with prognosis [17]. Inconsistently, our study identified four subgroups in PAAD using single-cell sequencing dataset, while both CAF_2 and CAF_4 subgroups showed poor prognosis, which were established by tallying up DEGs from the four groups into a single score. The distinct prognostic significance of CAF clusters might be attributed in part to the notable variation we observed in the TP53 signaling pathway between malignant and non-malignant cells within CAF_2 and CAF_4 subgroups, while such variation wasn’t evident in CAF_1 and CAF_3. Earlier research has indicated the involvement of TP53 mutation in the progression of PAAD tumors [25, 26].

The predictive efficacy of two CAF subgroups led us to develop a CAF-based risk signature consisting of seven genes. CD36, PHOSPHO1, PLD4, BCL11A, and RNF166 were the protective genes, while TLR1 and SERPINB5 were the risk genes. Notably, TLR1, BCL11A, PHOSPHO1, and SERPINB5 were all found to have SNV mutations in our research. Overall patient survival is correlated with the presence of a sense SNV mutation, which alters protein activity or function that leads to PAAD progression [27]. Despite the lack of evidence from independent research, our findings imply a possible role for SNV mutations in these risk genes in PAAD development. Additionally, we discovered a strong association between the seven genes and 60 pathways, some of which have previously been linked to carcinogenesis, including apoptosis [28], cytokine-cytokine receptor interaction [29], leukocyte transendothelial migration [30], and natural killer cell mediated cytotoxicity [31]. Therefore, these findings point us in the right path for future research into how the control of these risk genes manifests in PAAD.

Recent research indicates that tumor growth may be facilitated by CAFs’ interactions with the infiltrating immune cells in the TME [32]. In our investigation, we employed three distinct algorithms to assess the connections between seven prognostic genes and immune infiltration. The ESTIMATE analysis indicated significant positive correlations between four protective genes and one risk gene with stromal score, immune score, and ESTIMATE score. These results suggested a possible interaction between these genes and tumor immune microenvironment in PAAD and, by extension, that these genes could be valuable therapeutic targets for the disease. The tumor immune microenvironment encompasses diverse immune cells within tumor islets, collectively orchestrating the stage for an effective anticancer immune response in the TME. Tumor cells are able to escape immune cell surveillance when CAFs collaborate with these cells to create an immunosuppressive tumor microenvironment [33]. After calculating the immune cell abundance with CIBERSORT algorithm, multiple immune cells were found to correlate with the predictive genes, including naïve and memory B cells, CD8^+^ and CD4^+^ naïve T cells, and activated NK cells, which was consistent with the results of MCPcounter analysis. Compared to tumors without tertiary lymphoid structures (TLSs), PDAC tumors have an increased number of memory B cells [34]. Comparing TLSs from the TME to peripheral blood, there was a decrease in the number of IgD^+^ and IgM^+^/IgD^+^ naïve B cells, indicating that class switching occurred in the TME [35]. T cells play a role in tumor development, and therapies derived from T cells, like checkpoint blockade and chimeric antigen receptor T (CAR-T) cell therapy, have exhibited promising outcomes [36]. When a NK cell detects surface markers associated with oncogenic transformation, it may quickly and efficiently eliminate any number of neighboring cells. This unusual ability of NK cells can be used to stimulate both antibody and T cell responses, thus strengthening the case for their use as anticancer agents [37].

The majority of patients, however, exhibit either inherent or acquired resistance to immunotherapies [38]. According to our findings, the risk signature could identify individuals who were more likely to respond favorably to immunotherapies. Recent research has shown that mesothelial cell-derived CAFs can function as antigen-presenting cells by directly ligating and inducing antigen-specific regulatory T cells from naïve CD4^+^ T cells [39]. Therefore, CAFs might play a role in immune evasion within pancreatic cancer, offering insights into enhancing cancer immunotherapy strategies. Additionally, there was an observed positive correlation of M1 macrophages with the risk genes (TLR1 and SERPINB5), whereas M2 macrophages exhibited a positive correlation with the protective gene (BCL11A), hinting at a potential involvement of the risk genes in macrophage polarization. Studies revealed that LRRC15^+^ CAFs directly diminish CD8^+^ T cell activity and limit the response to checkpoint blockade through a TGFβ-dependent pathway [40]. Enhancements in patient survival and the response to immunotherapy could potentially result from the advancement of treatments aimed at re-establishing the balanced fibroblast state, thereby reducing the population of disease-promoting LRRC15^+^ myofibroblasts. Our results, on the other hand, suggested that a CAF-based signature might forecast a patient’s receptivity to anti-PD-L1 immunotherapy. These findings revealed previously unknown information on CAF’s function in modifying the TME and cancer niche. However, additional research is still needed to fully understand CAF-TIME communication’s function in PAAD.

Our study has a few limitations. Firstly, the development of CAF clustering and the CAF-based risk signature relied on retrospective data from open resources. It’s crucial to validate its effectiveness in other prospective and multi-center PAAD cohorts in the near future. Secondly, our focus was primarily on the potential predictive value of the CAF-based risk signature. Further research is necessary to unravel the mechanisms underlying the signature’s involvement in the progression of PAAD.

## CONCLUSIONS

In summary, our study provided a comprehensive characterization of PAAD CAF populations, identifying four distinct CAF subgroups exhibiting varied traits. The DEGs among these groups were found to be enriched in pathways like vascular smooth muscle contraction, focal adhesion, ECM-receptor interaction, and protein digestion and absorption. Leveraging two clusters significantly associated with PAAD prognosis, we formulated a CAF-based predictive risk signature composed of seven genes. This signature, when combined with clinicopathological parameters in a nomogram, demonstrated excellent prognostic performance for PAAD patient outcomes. Moreover, our findings suggested that this signature correlated with the immune landscape of PAAD, potentially serving as a tool to predict responses to PD-L1 blockade immunotherapy.

## Supplementary Material

Supplementary Figures
