# Supplementary material for: Revealing a cancer-associated fibroblast-based risk signature for pancreatic adenocarcinoma through single-cell and bulk RNA-seq analysis
Source: Aging (Albany NY). 2024 Sep 26;16(18):12525–42. doi: 10.18632/aging.206043 (PMC11466480; doi:10.18632/aging.206043)
Supplement: Supplementary Figures [file aging-16-206043-s001.pdf]

SUPPLEMENTARY FIGURES

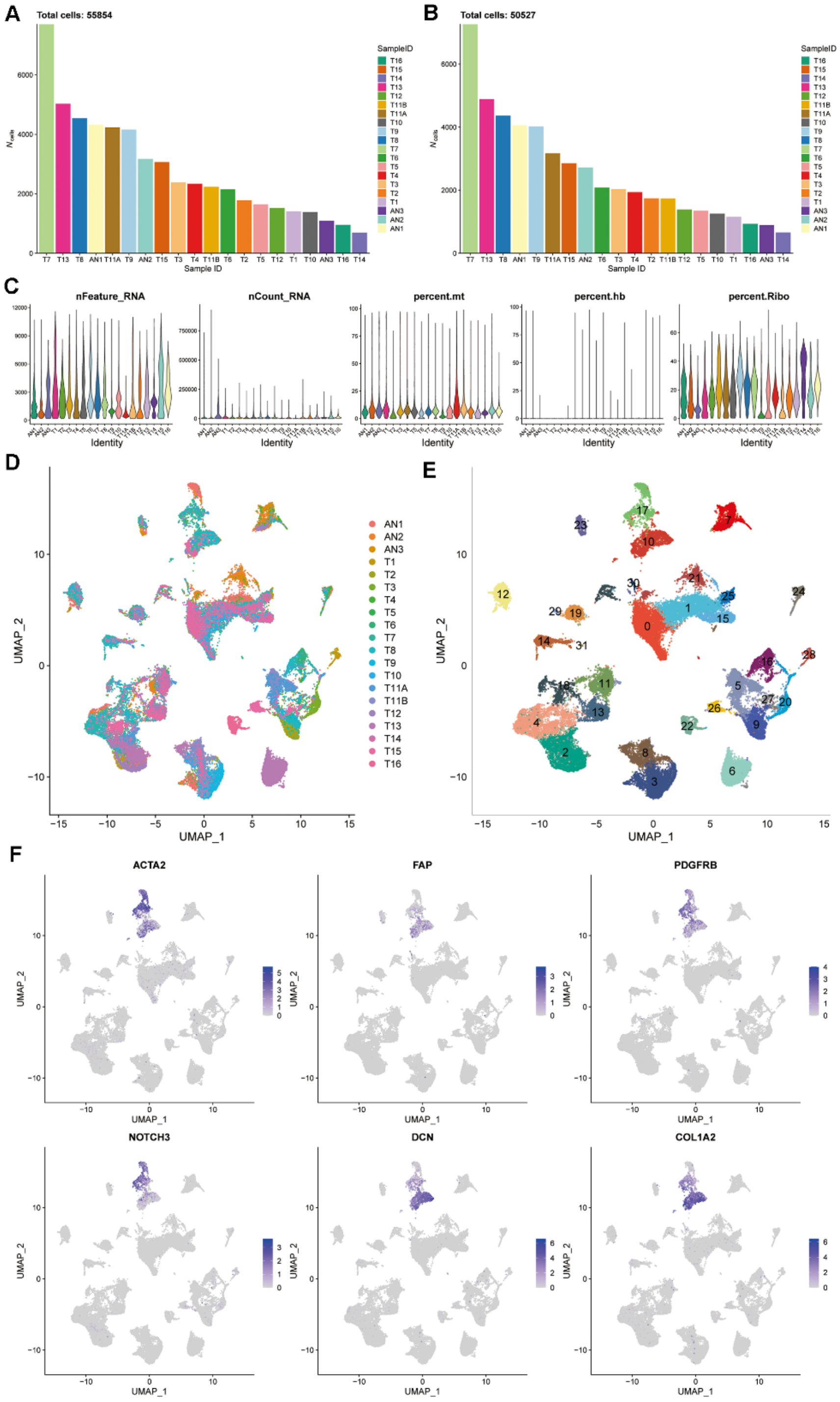

Supplementary Figure 1. Characterization of CAF populations utilizing scRNA-seq data from PAAD patients. (**A**) Total cell counts before filtering and (**B**) after filtering of single-cell data. (**C**) The quality control of scRNA-seq data of PAAD in individual samples before data filtering, including mRNA, UMI, mitochondrial content, hemoglobin content, and ribosome RNA content. (**D**) UMAP plot illustrating the distribution of 20 samples. (**E**) UMAP plot showcasing the distribution of subpopulations post-clustering of all cells. (**F**) UMAP plot displaying the expression of CAF marker genes (ACTA2, FAP, PDGFRB, NOTCH3, DCN, and COL1A2).

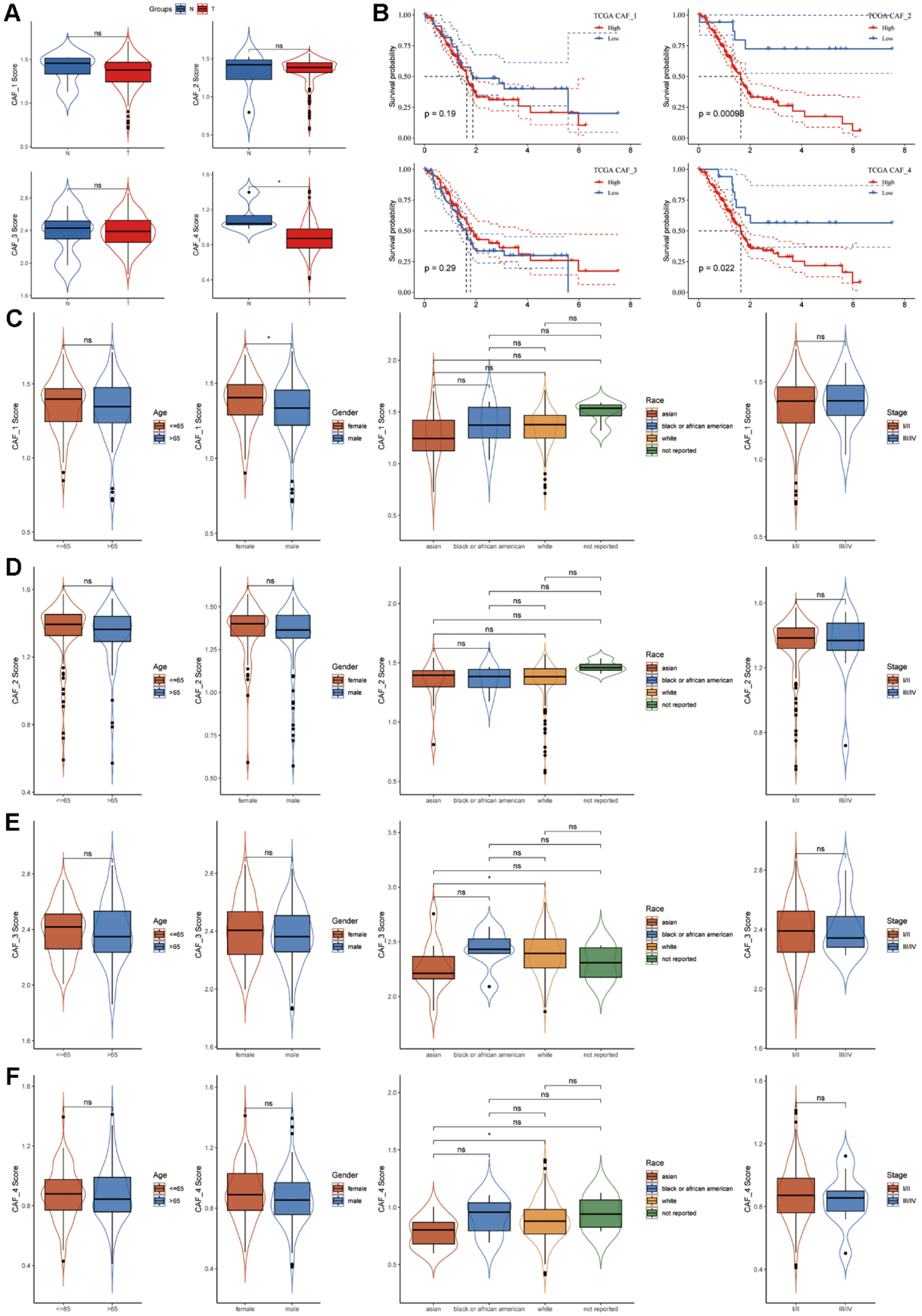

Supplementary Figure 2. Relationships between the four CAF clusters and PAAD patient prognosis. (**A**) Comparative analysis of four CAF scores within tumor and normal samples. (**B**) Kaplan-Meier curves depicting high and low CAF score groups across the four CAF clusters. Associations between (**C**) CAF_1, (**D**) CAF_2, (**E**) CAF_3, (**F**) CAF_4 clusters, and clinicopathologic factors, encompassing age, gender, race, and pathological stages.

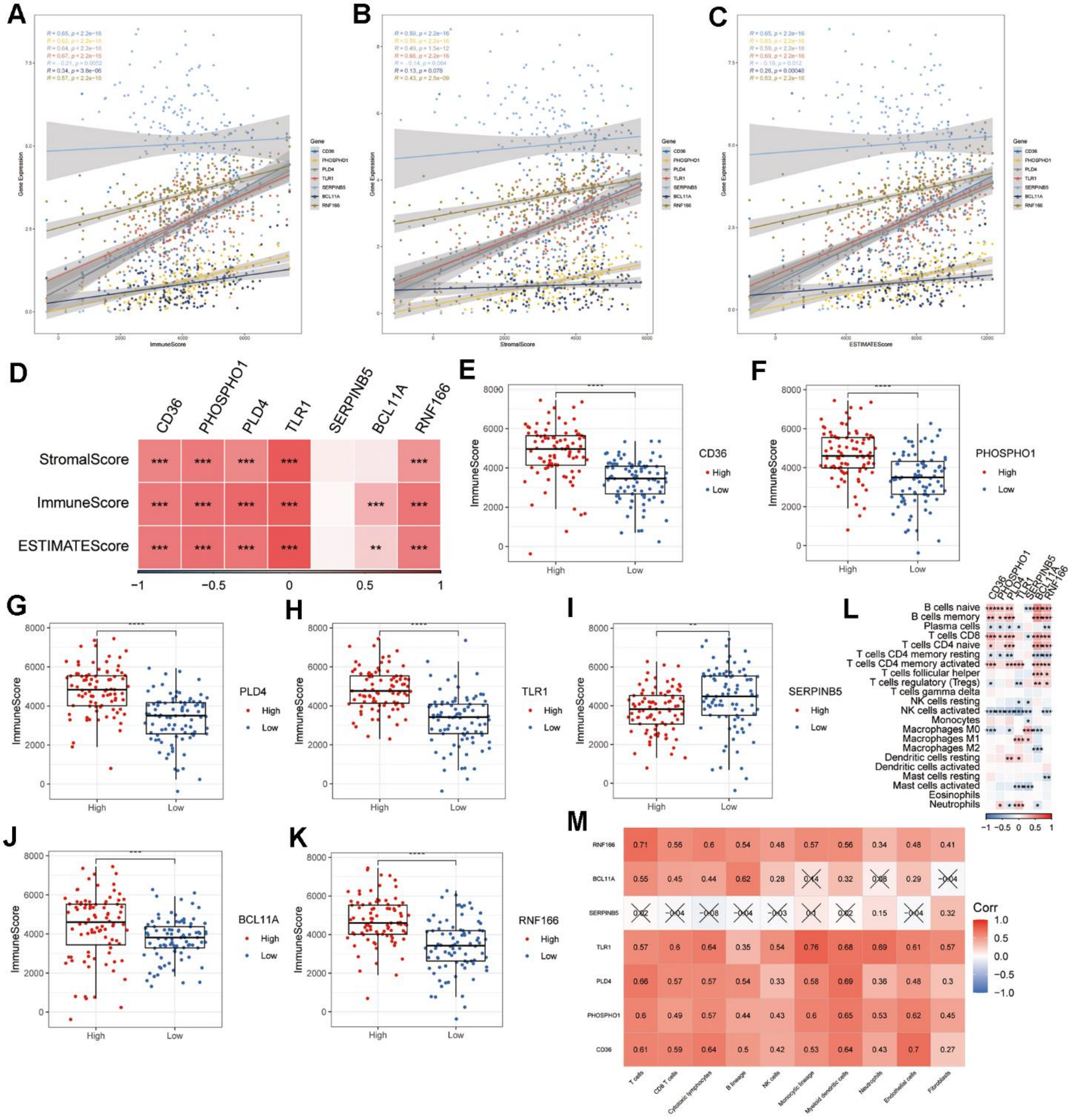

Supplementary Figure 3. Relationship between immune landscape and seven prognostic genes. Scatter plots depicting the correlation between the seven prognostic genes and (**A**) immune score, (**B**) stromal score, and (**C**) ESTIMATE score. (**D**) Heatmap displaying the correlation between the seven prognostic genes and stromal score, immune score, and ESTIMATE score. Comparison between high and low expression of (**E**) CD36, (**F**) PHOSPHO1, (**G**) PLD4, (**H**) TLR1, (**I**) SERPINB5, (**J**) BCL11A, (**K**) RNF166, and immune score (wilcox.test). (**L**) Correlation between pivotal prognostic genes and infiltrating immune cells computed via the CIBERSORT algorithm. (**M**) Correlation between key prognostic genes and infiltrating immune cells calculated using the MCPcounter algorithm.
